# T‐cell pseudolymphoma secondary to ixazomib for multiple myeloma

**DOI:** 10.1002/ski2.57

**Published:** 2021-06-30

**Authors:** M. Haq, Y. Reyal, N. Tiffin, S. Szakacs, L. Ferguson

**Affiliations:** ^1^ Faculty of Medicine St George’s, University of London London UK; ^2^ Department of Haematology St George’s Healthcare NHS Trust London UK; ^3^ South West London Pathology St George’s Healthcare NHS Trust London UK; ^4^ Department of Histopathology William Harvey Hospital, East Kent Hospitals University NHS Foundation Trust Ashford UK; ^5^ Department of Dermatology St George’s Healthcare NHS Trust London UK

## Abstract

We present a case of a 54‐year‐old male with multiple myeloma (MM) who presented with widespread pruritic erythematous lesions following ixazomib treatment. This occurred after his third cycle of treatment with ixazomib, thalidomide and dexamethasone and was controlled by potent steroids and temporary cessation of ixazomib. The strong correlation between the timeline of the rash, ixazomib treatment and subsequent cessation led to a diagnosis of a drug‐induced rash. Skin biopsy histology, immunochemistry and the absence of monoclonal T‐cell receptor gene rearrangement further confirmed the diagnosis of a T‐cell pseudolymphoma secondary to ixazomib. Ixazomib is an oral proteasome inhibitor used in the treatment of MM. Other proteasome inhibitors have been reported to trigger cutaneous adverse effects. However, to our knowledge, this is the first report of pseudolymphoma following proteasome inhibitor use. Dermatologists should be aware of this potential effect and the possible management pathways such as cessation and dose reduction.

## Graphical abstract

We report a 54‐year‐old male with multiple myeloma who presented with widespread pruritic erythematous lesions following ixazomib, a type of proteasome inhibitor. Skin biopsies, immunochemistry, and monoclonal TCR gene rearrangement confirmed the diagnosis of a T cell pseudolymphoma secondary to ixazomib. To our knowledge, this is the first report of pseudolymphoma following proteasome inhibitor use.

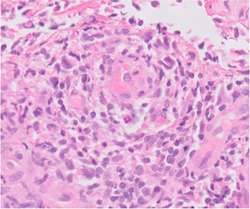

**What is already known about this topic?**

- Proteasome inhibitors have been reported to cause cutaneous effects, such as leukocytoclastic vasculitis, in some patients.
- Cutaneous pseudolymphomas are rare and have many potential causes.

**What does this study add?**

- To our knowledge, this is the first report of cutaneous pseudolymphoma following proteasome inhibitor use.
- Possible management of T‐cell pseudolymphoma via temporary cessation or dose reduction techniques.

A 54‐year‐old black British male developed multiple painful, pruritic lesions which became widespread over 5 days, starting on his face, progressing to trunk and limbs. He had no associated systemic symptoms. He was diagnosed with multiple myeloma (MM) in 2012. After initial treatment response, relapse in 2018 was managed with four cycles of ixazomib, thalidomide and dexamethasone (ITD), with a plan for subsequent stem cell transplantation. The rash developed during the third cycle of ITD, following his ninth dose of ixazomib. Additional past medical history included discoid lupus erythematosus and extensive idiopathic vitiligo.

On examination, there were multiple, tender, discrete 0.5–1 cm indurated erythematous papules and nodules over his neck, finger, leg, back and trunk (Figure 1).

**FIGURE 1:**
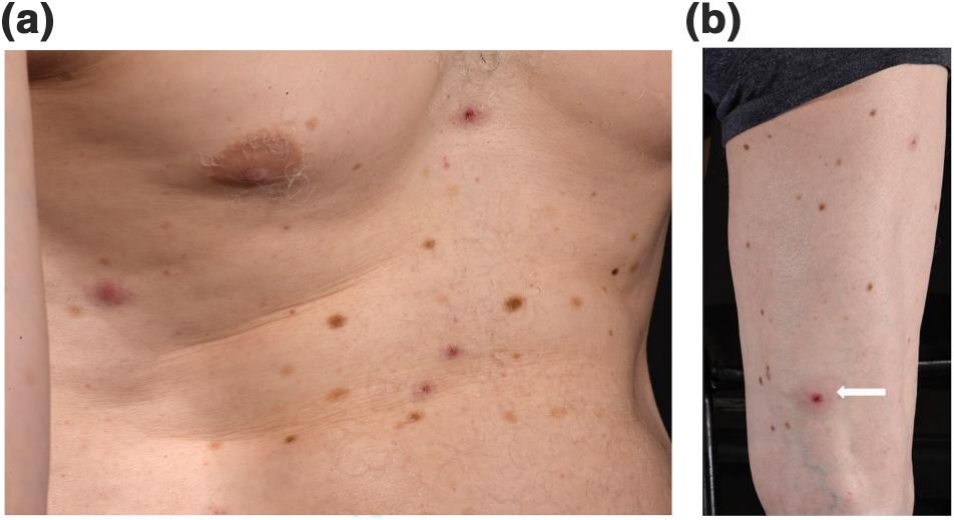
(a) Well‐circumscribed erythematous macules which have a slightly targetoid appearance which measured 5–10 mm. Papules can be seen on both the right side of the chest as well as more indurated papules centrally, on a background of widespread depigmentation due to long‐standing vitiligo. (b) Lesion on the thigh with a central ulcerated dark erythematous papule and surrounding erythema with induration

Skin biopsy demonstrated an atypical angiocentric lymphoid infiltrate throughout the dermis, extending into subcutaneous fat (Figure 2). Immunochemistry confirmed T‐cell lineage, predominantly CD8 positive. Stains for CD20, CD30, anaplastic lymphoma kinase, epithelial membrane antigen and EBV encoded small RNA for Epstein–Barr virus were negative. No monoclonal T‐cell receptor gene rearrangement was seen, which combined with the strong temporal correlation of the rash with treatment, made T‐cell lymphoma unlikely. Staining to exclude a gamma delta lymphoma and EBV screen were negative.

**FIGURE 2:**
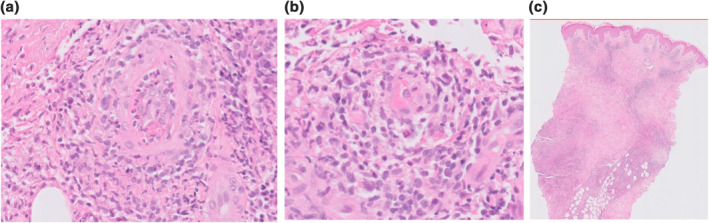
(a) Histology: H&E stain shows a vessel surrounded by large, atypical lymphoid cells in an angiocentric pattern. The endothelium of the vessel is very swollen indicating early vascular damage, which combined with the atypical lymphoid infiltrate imitates an angiocentric lymphoma hence the term angiocentric pseudolymphoma. Level of magnification: ×20 objective magnification. (b) H&E stain shows a smaller vessel showing vascular damage. Level of magnification: ×20 objective magnification. (c) Low‐power H&E stain showing a superficial and deep infiltrate within the dermis and subcutis. H&E, haematoxylin and eosin

Ixazomib was temporarily stopped and super‐potent topical steroids and chlorpheniramine administered. Improvement occurred within 7 days of ixazomib cessation and resolution within 13 days, at which point ixazomib was reintroduced with a 25% dose reduction. Subsequent recurrence of the rash occurred during cycle 4 of ITD after two further doses of ixazomib (at day 14), which supported our suspicion that this was drug‐induced, but as the rash was mild no additional dose modification was required. He subsequently underwent an autologous stem cell transplant and did not receive further ixazomib. We concluded the lesions represented cutaneous T‐cell pseudolymphoma secondary to ixazomib.

Cutaneous pseudolymphomas represent a rare reactive process that involves lymphocytic infiltrates which ultimately mimic cutaneous lymphomas. The infiltrate's imitation is based on clinical as well as histopathological findings.^1^ The most common form of cutaneous pseudolymphoma is nodular pseudolymphoma which consists of B‐cell, T‐cell and mixed pseudolymphomas.^1^ Recognized triggers of cutaneous pseudolymphomas include lymphomatoid drug reactions, lymphomatoid contact dermatitis and infections including Borrelia‐associated T‐pseudolymphoma.^1^ Drugs known to cause cutaneous pseudolymphomas include antihypertensive, anticonvulsants, anxiolytics and antipsychotics.^1^ To our knowledge, this is the first report of ixazomib triggering a T‐cell pseudolymphoma.

Ixazomib is an oral proteasome inhibitor (PI) used in the treatment of MM.^2^ Reported toxicities include thrombocytopenia, peripheral neuropathy and unclassified rashes (seen in 36%).^2^ Ixazomib was reported to cause cutaneous necrotizing vasculitis in one 66‐year‐old with MM following 33 days of treatment.^3^ Here, after the second cycle, the patient developed multiple urticated plaques, managed with temporary ixazomib cessation followed by dose reduction. Similarities were seen clinically between our patient and this 66‐year‐old patient as both patients had similar looking purpuric papules, with an almost urticarial appearance in similarly situated areas of the body: neck, chest and back. However, biopsies in this 66‐year‐old patient revealed a mixed acute and chronic granulomatous necrotizing vasculitis with eosinophils present as well as a vacuolar interface dermatitis.^3^ Biopsies from our patient revealed a more angiocentric lymphoid infiltrate that was present in the dermis as well as the subcutaneous fat and had a confirmed T‐cell lineage, hence histologically both cases differ.

Older PIs also have reported cutaneous effects. Bortezomib triggered a leukocytoclastic vasculitis in a female patient with MM,^4^ responsive to corticosteroid treatment, which recurred on drug reintroduction. Additionally, during three phase II trials in patients with non‐Hodgkin lymphoma, bortezomib was seen to cause an erythematous maculopapular rash in 26 patients over the three trials. Biopsies revealed a non‐necrotising cutaneous vasculitis, with no evidence of lymphoma and a perivascular lymphocytic infiltrate was seen. In these cases, it was observed that the rash was not dose‐dependent as patients on different doses over the three trials developed the rash.^5^ In addition, another case report of bortezomib‐induced leukocytoclastic vasculitis was seen in a 55‐year‐old male with MM.^6^ This patient had similar findings to our patient, with a purpuric rash starting on day 9 of the third cycle of treatment and occurring on the patient's trunk, back, extremities and face. However, a biopsy of the rash showed leukocytoclastic vasculitis in the dermis as well as extending into the superficial subcutaneous fat with vessel wall destruction and accumulation of inflammatory cells.^6^ Histologically this differed from our presented case as a lymphoid infiltrate of T‐cell lineage was seen on biopsy with no vessel wall damage. It was also found that the 55‐year‐old male patient being treated with bortezomib had increased levels of tumour necrosis factor alpha, interleukin 6 and C‐reactive protein in his serum that researchers hypothesized may have led to the bortezomib‐induced cutaneous leukocytoclastic vasculitis, which was not seen in our case.^6^

Interestingly, ixazomib is being trialled for systemic lupus erythematosus (SLE), in the hope that via a reduction in plasma cells, SLE autoantibodies will be reduced.^7^ We are uncertain if our patient's autoimmune comorbidities may have been relevant to developing pseudolymphoma.

This report summarizes a rare cutaneous drug reaction to ixazomib. This is the first report of pseudolymphoma with PIs. Dermatologists should be aware of this due to the increasing use of PIs, and management options include temporary cessation or dose reduction.

## CONFLICTS OF INTEREST

The authors declare that there is no conflict of interest.
